# Relationship between mothers’ enjoyment and sedentary behavior and physical activity of mother–child dyads using a movement-to-music video program: a secondary analysis of a randomized controlled trial

**DOI:** 10.1186/s12889-020-09773-4

**Published:** 2020-11-04

**Authors:** Pipsa P. A. Tuominen, Jani Raitanen, Pauliina Husu, Riitta M. Luoto, Urho M. Kujala

**Affiliations:** 1 University of Jyväskylä, Faculty of Sport and Health Sciences, PO Box 35, FI-40014 Jyväskylä, Finland; 2 Tampere University of Applied Sciences, Degree program of Physiotherapy, Tampere, Finland; 3 UKK Institute for Health Promotion Research, Tampere, Finland; 4 Tampere University, Faculty of Social Sciences (Health Sciences), Tampere, Finland; 5 Tampere University, Faculty of Medicine and Biotechnology, Tampere, Finland

**Keywords:** Device-based measurements, Accelerometer, Enjoyment in sport, Movement-to-music, Music-based intervention, Motivation

## Abstract

**Background:**

Parental support and participation in physical activity (PA) with children and parents’ acting as a role model for less sedentary behaviors (SB) are critical factors for children’s healthier lifestyle. The purpose of the study was to assess the relationship between mothers’ enjoyment and participants’ sedentary behavior (SB) and physical activity (PA) as a secondary analysis of a randomized controlled trial (RCT) using data from Moving Sound RCT in the Pirkanmaa area of Finland.

**Methods:**

The participants were 108 mother–child dyads (child age 5–7 years) who completed the eight-week exercise intervention using a movement-to-music video program in their homes. Mothers’ enjoyment was examined using a modified version of the enjoyment in sport questionnaire. The proportion of SB, standing, light PA, moderate-to-vigorous PA, and Total PA were derived from accelerometers at baseline and during the final week of the intervention. Analyses were performed using linear mixed-effect models for (1) intervention and control groups, (2) groups based on mothers’ enjoyment.

**Results:**

The results highlighted that mothers’ enjoyment of exercise with their children was overall high. Although there was no difference between the intervention and control groups, mothers in the intervention group increased their enjoyment during the intervention (*p* = 0.007). With mothers’ higher enjoyment at baseline, children’s light PA increased (*p* < 0.001), and with mothers’ lower enjoyment, children’s SB increased (*p* = 0.010). Further, if mothers’ enjoyment decreased during the study, their own LPA increased (*p* = 0.049), and their children’s SB increased (*p* = 0.013). If mothers’ enjoyment remained stable, children’s light PA (*p* = 0.002) and Total PA (*p* = 0.034) increased.

**Conclusions:**

In this RCT, no differences were found between the intervention and control groups or groups based on mothers’ enjoyment, possibly due to the low power of the study. However, mothers’ enjoyment of exercise with their children increased within the intervention group, and mothers’ enjoyment influenced children’s SB and PA. For future studies, it would be essential to focus on children’s enjoyment and factors behind the behavior change.

**Trial registration:**

The study is registered at ClinicalTrials.gov, registration number NTC02270138, on October 2, 2014.

**Supplementary Information:**

The online version contains supplementary material available at 10.1186/s12889-020-09773-4.

## Background

Health professionals and researchers encourage parents to be active with their children. However, results relating to the influence of parental behavior on children’s PA are inconsistent [1]. Parents’ participation in physical activity (PA) with their children and parental support for children’s PA have been reported to be one of the critical factors for young children’s moderate-to-vigorous physical activity (MVPA) in earlier intervention studies [1–3]. In addition, parental support is positively and strongly associated with children’s PA through informational, emotional, appraisal, and instrumental mechanisms, among others [1]. Moreover, parents as a role model for their child’s sedentary behavior (SB) has received increasing interest in several studies [4, 5]. How do parents and their children adopt and adhere to a healthier lifestyle?

In lifestyle interventions, SB is often associated with television viewing [4–6], and a larger amount of parents’ sedentary time is associated with increased risk of higher sedentary time for children [5, 6], but the factors influencing SB are multi-dimensional and complex [4]. It is also important to keep in mind that SB includes all lying, reclining, and sitting behaviors during waking hours, not only TV or screen viewing. Furthermore, by changing passive screen time to activity with plays and games, it might be possible to decrease sedentary time. PA interventions targeted at both parents and children may also generate a reduction in SB among children [7].

In the home environment, music is used mainly for entertainment, including TV and video watching [8]. However, studies have shown that PA programs with music may motivate children to engage in PA [9]. Within families, mothers are more likely to choose music for background purposes, while children themselves select music as the central part of activities, such as general and musical play, wherein music is listened to, sung, or played with an instrument [8].

Parents’ motivation to exercise has been found to increase their MVPA if they perceived exercise as personally essential or valuable to them [3]. Self-determination theory [10], where internal forces, such as competence, relatedness, and autonomy, are strong determinants of behavior, has been used to explain changes in PA and SB interventions [3]. Enjoyment in sport (EIS) and personal investments have been identified as a considerable portion of sport commitment [11]. Participants’ enjoyment is used to promote behavioral change because it is given as a reason for greater motivation and commitment [12–15]. Enjoyment as a part of internal motivation has been defined as reflecting feelings such as pleasure, liking, and fun [11]. Moreover, parents have reported benefits for PA from co-participation with their children, such as spending quality time together, improving children’s general health and well-being, and the development of physical skills [16]. However, even if different aspects of motivation have been widely studied, less is known about the relationship between parental enjoyment of exercise with their child and children’s PA and SB.

This paper reports mothers’ enjoyment of performing exercise with their child as an outcome of the Moving Sound randomized controlled trial (RCT, ClinicalTrials.gov, NTC02270138). As a secondary analysis, we were interested in the relationship between mothers’ enjoyment and SB as well as PA. The rationale for this arises from attentional involvement through the suggestion that a given task (exercising with the child by using the movement-to-music video program) is associated with positive experiences, and focused attention is intrinsically rewarded [17, 18]. In addition, mother–child interactions and communication may benefit from shared musical activities [19], and thus increase the enjoyment and amount of exercises.

## Methods

### Aims and hypothesis of the study

The study is based on the Moving Sound RCT (*n* = 228 mother–child dyads, child age 5–7 years), which assessed the effects of a movement-to-music video exercise program in the home environment on the SB and PA of mothers and their children in an eight-week intervention. These results have been reported previously [20, 21]. In brief, no statistically significant differences between the intervention and control groups were found in device-measured SB or PA, or in self-reported screen time. The children who stayed at home instead of attending daycare or preschool had more SB and less MVPA than those who were at daycare or preschool. Furthermore, within the intervention group, the children whose mothers had music-based hobbies had a higher probability of increasing their light PA (LPA) during the intervention, but not their MVPA compared to the children whose mothers did not have music-based hobbies. Mothers without music-based hobbies had a higher probability of increasing their LPA and MVPA and decreasing their SB than did mothers with music-based hobbies. It was also found that mothers without music-based hobbies were more likely to belong to the highly motivated by music group compared to mothers with music-based hobbies (29% vs. 18%). The number of mothers who thought that the motivational effect of music was neutral was slightly less than one-third in both groups.

The main aim of the current study was to assess the relationships between the use of the exercise program and mothers’ enjoyment of performing exercises with their child during an eight-week intervention. The secondary purpose was to explain differences in exercise activity (i.e., adherence as the number of exercise sessions) between mothers and children within the intervention group and assess the relationships between mothers’ enjoyment and their own and their children’s device-measured PA and SB.

Our hypotheses were as follows:

1. Mothers’ enjoyment is at the same level at baseline in both the intervention and control groups, and mothers in the intervention group are more likely to show increased enjoyment than are mothers in the control group during the intervention.
2. Regarding the intervention group, on average, mothers who exercise as much as instructed using the video program (i.e., adherent group) increase their enjoyment more compared to those who do not use the video (i.e., non-adherent group).
3. If children exercise as much as instructed using the video program (i.e., adherent group), their mothers show increased enjoyment more than do the mothers of those children who do not use the video (i.e., non-adherent group).
4. As a supplementary analysis, mothers who have higher enjoyment at the baseline are physically more active and have less SB than the mothers who score low on enjoyment. Moreover, children whose mothers have higher enjoyment are physically more active and have less SB than the children whose mothers score low on enjoyment.
5. Mothers who show increased enjoyment are more likely to decrease their SB and increase their PA compared to those mothers whose enjoyment remains stable or decreases.
6. Children whose mothers show increased enjoyment are more likely to decrease their SB and increase their PA compared to those whose mothers show stable or decreased enjoyment.

### Participants

Mother–child dyads for the Moving Sound RCT (*n* = 228) were recruited between November 2014 and January 2016 from the cohort of NELLI: Pregnancy as a window to the future health of mothers and children, a seven-year follow-up of a gestational lifestyle intervention in the Pirkanmaa area of Finland [20, 22]. Participants in the current study were mother–child dyads (*n* = 108) who had acceptable accelerometer measurements (having at least four measurement days during both the baseline and the final intervention week and at least 10 h per day) and who answered the questions about mothers’ enjoyment of exercising with their child at both the beginning and the end of the intervention. Mother–child dyads who withdrew (*n* = 25) or did not meet these criteria (*n* = 95) were excluded.

### Design and intervention

All mothers (*n* = 108) and children (*n* = 108) were instructed to use a tri-axial hip-worn accelerometer (Hookie AM20, Traxmeet Ltd., Espoo, Finland) every day during waking hours for weeks 1 (baseline/reference week), 2 (the first intervention week), and 8 (the final intervention week). For the same weeks, mothers were instructed to complete exercise diaries for themselves and their children. Mother–child dyads in the intervention group (*n* = 50) were instructed to use the movement-to-music video program DVD every other day from the beginning of Week 2 to the end of Week 8. The program was based on PA recommendations from 2008 and included three separate exercise programs, each lasting 10 min [22]. In brief, the music (children’s rock, Latin, and folk) and videos for the program were produced by the music education students from Sibelius Academy and pretested for the motivational qualities by a panel of female physiotherapists. The videos included exercises to improve and maintain aerobic fitness, muscle strength, balance, and coordination. Each song had its own movements, which were performed to the beat of the music. For the suitable amount of exercises for themselves, each mother–child dyad could use the videos individually or consecutively. Mother–child dyads in the control group (*n* = 58) were instructed to behave and exercise as they usually do.

### Measures

Mothers’ enjoyment was the primary outcome of the present study, and it was measured using the Finnish version [23] of the Enjoyment in Sport (EIS) questionnaire [11] before the baseline week and after the study period (Week 8). Questions were modified to be appropriate for exercising with children; for example, the statement “I like exercising” was changed to “I like exercising with a child” [22]. The EIS questionnaire included four statements for liking, enjoying, having fun, and happy playing, each one rated with a number from 1 (strongly disagree) to 5 (strongly agree) [11, 23]. The range of total scores for the mother’s enjoyment was 4–20, with the lowest score indicating minor enjoyment and the highest score indicating great enjoyment of exercising with the child.

Device-based measurements were performed with the hip-worn accelerometer (Hookie AM20, Traxmeet Ltd., Espoo, Finland), which collected and stored the tri-axial acceleration signal in raw mode [24]. Mean amplitude deviation values (6-s epochs) were converted to METs (metabolic equivalent, 3.5 ml/kg/min of oxygen consumption), and intensity was calculated as epoch-wise MET values [24]. Parameters analyzed included SB (< 1.5 MET, i.e., lying, reclining, and sitting down), standing (SS < 1.5 MET), light PA (LPA 1.5–2.9 MET), moderate-to-vigorous PA (MVPA ≥6.0 MET), and Total PA as a proportion of measurement time (meaning accelerometer wearing time) [25, 26]. Mothers and children were instructed to use the accelerometer during waking hours for seven consecutive days on each measurement week, excluding water activities. Non-wear time was calculated since the accelerometer did not detect the acceleration signal for 30 min. Mothers and children who used accelerometers for at least 4 days during both the baseline and the last intervention week and at least 10 h per day were included in the analysis. The mothers were also given diaries for the same weeks for themselves and for the child, in which they recorded their working hours and the child’s daycare or preschool hours, exercises, and the time engaged in PA. The data of completed exercises with the video program were collected from diaries. All the acceptable data from accelerometer measurements were used for analysis and the use of the video program was not separated from the data. Results regarding device-based measurements have been reported previously and described briefly at the beginning of the methods section [20, 21, 27].

### Statistical analysis

The required sample size was calculated before the study using a two-sample means test with a power of 80% and a two-sided alpha of 5% [22, 27]. As a relation to mothers’ sedentary time, the effect size was 0.500, and the estimated sample size for the study was 63 mother–child dyads per group [27].

Baseline characteristics were reported as means and standard deviations (SD) for continuous variables and as frequencies and percentages for categorical variables. A Mann-Whitney U-test and a Fisher exact test were used for the differences of background characteristics between the groups at the baseline. A linear mixed-effects model (LME) was used to analyze the differences in enjoyment within and between the intervention and the control groups, use of the video within the intervention group, and for differences in device-measured outcomes between enjoyment groups. LME models for analysis were tested for potential confounding factors. Potential confounding factors were included in the analyses by adding them one by one to the model to see if the estimates for interaction term changed in the primary outcomes. The change of the estimates for interaction terms was not essential, and therefore non-adjusted models were used. However, LME models related to SB and PA were adjusted for the measurement time. For analysis purposes, mothers and children who used the video program during the final week of the intervention were considered to have performed exercises according to the instructions (i.e., adherent group). All the other intervention mothers and children were included in the group who did not use the video program as instructed within the intervention group (i.e., non-adherent group). For the final analysis, mothers were classified to either the higher enjoyment group (i.e., scores ≥18) or the lower enjoyment group (i.e., scores < 18), based on the median of scores at the baseline. Changes in the enjoyment scores from baseline to the end were modified into tertiles (increased, stayed stable, or decreased). Outliers were removed prior to the analysis if standardized values (*z* score) were less than − 3.30 or greater than 3.30. A two-tailed significance level of 0.05 was used for the analyses.

In the lost to follow-up analysis, Fisher’s exact test was used for dichotomous variables (group, gender of the child, being in work, the child staying in daycare or preschool), and the independent samples *t* test was used for continuous variables (age, BMI, perceived health) to determine whether there were differences between those who were excluded from the study compared to those who were included. Analyses were performed using Stata 15.1 and SPSS 24.0.

## Results

### Background characteristics of the intervention and control groups

The background characteristics regarding the intervention and control groups are presented in Table 1. Any differences between the intervention and control groups were not found except in marital status, according to which 90% of the intervention mothers and 100% of the control mothers were married or cohabited (*p* = 0.019).

**Table 1:** Background characteristics of the participants in the intervention and control groups

		Intervention	Control		Difference between groups, *p* value
	*n*	mean (SD) / *n* (%)	*n*	mean (SD) / *n* (%)	
Mothers					
Age (in 2015)	50	36.4 (4.6)	58	38.2 (5.1)	0.059^a^
Married or cohabited	50	45 (90.0)	58	58 (100.0)	**0.019**^b^
Number of children	50	2.5 (1.1)	57	2.4 (0.9)	0.88^a^
Employment, at work	50	40 (80.0)	58	51 (87.9)	0.30^b^
BMI	50	27.1 (5.2)	58	27.5 (5.3)	0.80^a^
Perceived health in VAS (0–100)	49	75.9 (16.3)	58	74.1 (13.3)	0.26^a^
Children					
Age	50	6.6 (0.5)	58	6.5 (0.5)	0.18^a^
Gender, girls	50	23 (46.0)	58	34 (58.6)	0.25^b^
BMI-for-age	50	22.2 (4.0)	56	22.2 (4.1)	0.84^a^
Daycare or preschool	50	47 (94.0)	58	55 (94.8)	1.00^b^

Data are presented as mean ± standard deviation (SD) or the number of participants (*n,* %)

^a^ A Mann-Whitney U-test

^b^ A Fisher’s exact test

### Hypothesis 1: mothers’ enjoyment of performing exercises with their child

**Fig. 1:**
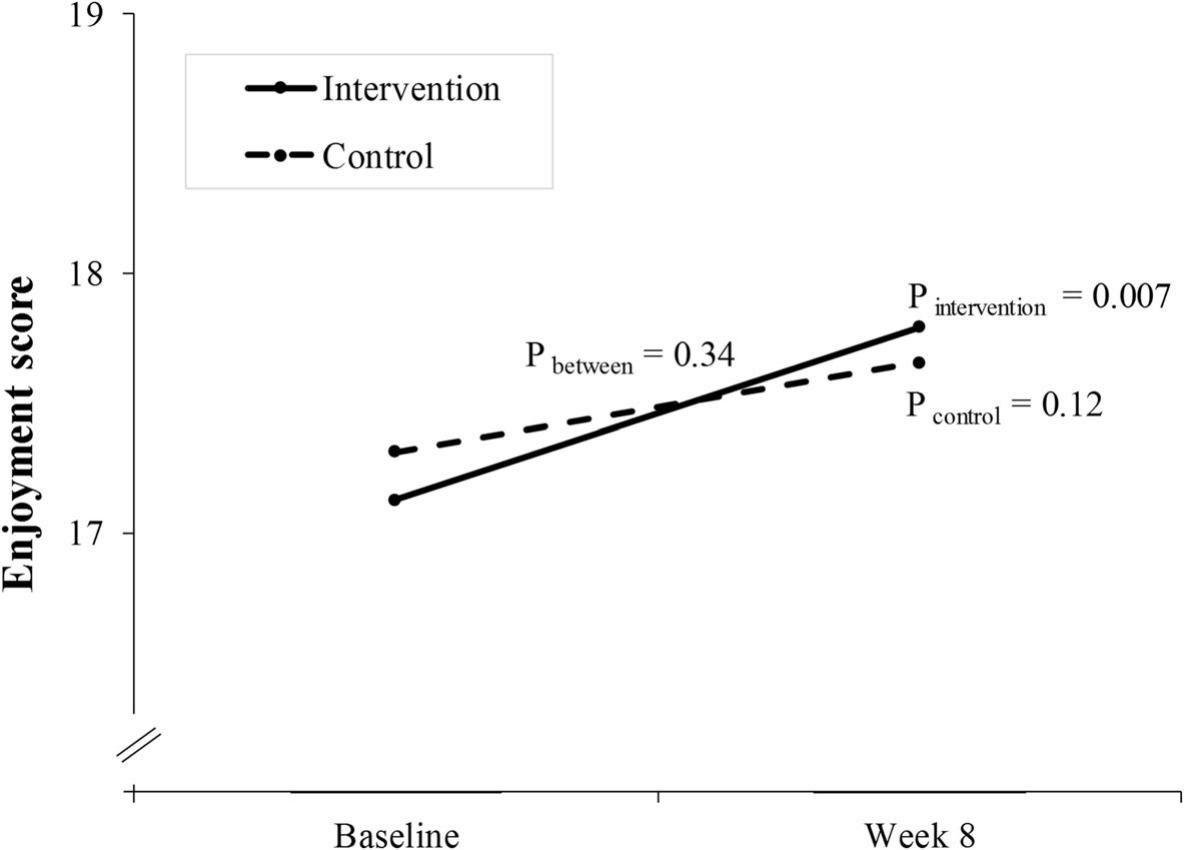
Mean motivation scores at the baseline and the last intervention week, and *p* values for within- and between-group changes for intervention and control mothers

The mothers’ enjoyment with the child was, on average, 17.1 (3.0) in the intervention (*n* = 48) and 17.3 (2.9) in the control group (*n* = 58) at the baseline, and it did not differ between the groups. As was hypothesized, mothers in the intervention group scored higher on enjoyment at the end of the study than they did at the baseline (*p* = 0.007, Fig. 1). Additionally, mothers in the control group scored higher on enjoyment at the end, but the difference within the group or between the groups was not statistically significant.

### Hypotheses 2 and 3: the use of the video program and enjoyment

Regarding the intervention group, eight mothers and nine children out of 48 mother–child dyads reported use of the movement-to-music video program during the final intervention week. At the baseline, mothers’ enjoyment was higher among those mothers who used the video as instructed during the final intervention week (the adherent group) when comparing to those who did not use the video (the non-adherent group as reference). However, the difference was not statistically significant (Table 2). Mothers in the non-adherent group scored higher on enjoyment at the end (*p* = 0.049), and so did mothers in the adherent group, but the latter result did not achieve statistical significance. The difference between groups was not statistically significant either, indicating that the results do not support the second hypothesis.

**Table 2:** Differences in enjoyment at baseline and change in the intervention mothers’ enjoyment scores within and between the groups who used (adherent group) or did not use (non-adherent group as reference) the video (estimates, 95% confidence intervals, and *p* values from a linear mixed-effects model)

	Estimate (95% CI)	*p* value
Mothers (*n* = 48)		
Difference in enjoyment at baseline (ref = non-adherent group)	1.50 (− 0.56 to 3.56)	0.15
Change in time, non-adherent group	0.09 (0.0002 to 0.17)	**0.049**
Change in time, adherent group	0.14 (−0.05 to 0.33)	0.14
Adherence effect (ref = non-adherent group)	0.06 (−0.15 to 0.27)	0.59
Children (*n* = 48)		
Difference in mothers’ enjoyment at baseline (ref = children’s non-adherent group)	1.21 (−0.75 to 3.18)	0.23
Change in time, non-adherent group	0.07 (−0.01 to 0.16)	0.094
Change in time, adherent group	0.19 (0.01 to 0.37)	**0.036**
Adherence effect (ref = children’s non-adherent group)	0.12 (−0.08 to 0.31)	0.25

Moreover, if mothers in the intervention group scored higher on enjoyment at the baseline, their children were more likely to use the video (Table 2). However, the difference between the groups was not statistically significant. In both groups of children, mothers’ enjoyment of performing exercises with them increased during the intervention. The relationship was statistically significant (*p* = 0.036) only if the children used the music video. The results indicate that the findings partly support the third hypothesis.

### Hypothesis 4: the relationship between mothers’ enjoyment and children’s SB and PA

**Fig. 2:**
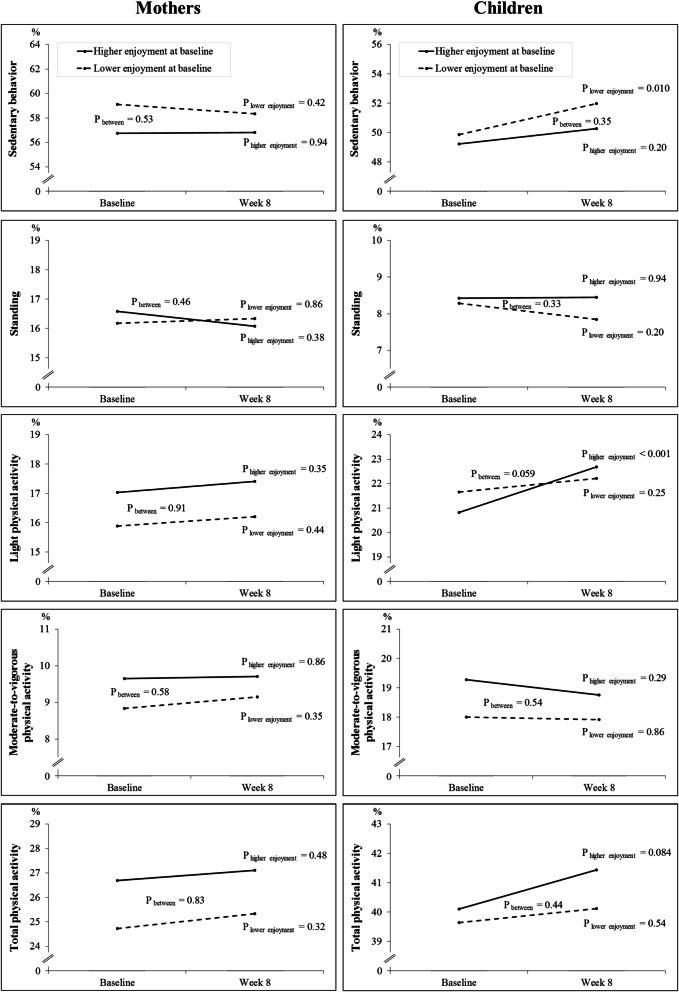
The relationship between mothers’ baseline motivation and mothers’ and their children’s sedentary behavior and physical activity

The relationship between mothers’ higher (*n* = 55) or lower (*n* = 53) baseline enjoyment (cut point = 18, based on median values of enjoyment scores) and their own and their children’s SB and PA are presented in Fig. 2. At the baseline, any statistically significant differences in SB or PA were not found among mothers or children. Among mothers, no statistically significant differences between or within the groups from baseline to the end of the eight-week intervention were found, either. However, statistically significant within-group differences from baseline to the end were found in increased SB (*p* = 0.010) among children whose mothers had lower enjoyment. Further, children’s LPA increased over time among those children whose mothers had higher enjoyment (*p* < 0.001), but statistical significance between the groups was narrowly missed (*p* = 0.059). A within-group increase of Total PA among children whose mothers had higher enjoyment approached near significance (*p* = 0.084). Thus, the fourth hypothesis was partly supported by the results.

### Hypotheses 5 and 6: the relationships between changes in mothers’ enjoyment and SB and PA

Among mothers, no statistically significant differences relating to changes in enjoyment and device-based outcomes were found, except for an increase in LPA (*p* = 0.049) among those mothers whose enjoyment decreased during the study (Supplementary Table 1). Narrowly missed significance (*p* = 0.055) was also found in the increase of Total PA among these mothers. Thus, the findings do not support the fifth hypothesis.

Children with mothers who reported increased enjoyment during the intervention had more SB and less MVPA at the baseline compared to the children with mothers whose enjoyment decreased (Supplementary Table 2), even if the first difference was not statistically significant (*p* = 0.065 and *p* = 0.037, separately). Moreover, if mothers’ enjoyment increased, children increased their proportion of LPA over the study period. However, the finding was not statistically significant (*p* = 0.064), and the difference in change between groups was not found. Children with mothers whose enjoyment remained stable increased their LPA (*p* = 0.002) and Total PA (*p* = 0.034) over the study period, but the difference in change between the groups was not significant, either. Furthermore, children with mothers whose enjoyment decreased, increased their SB (*p* = 0.013) over time, but the difference between the groups was not found. Thus, the findings do not support the sixth hypothesis.

### Lost to follow-up analysis

During the original Moving Sound RCT (*n* = 228), 25 mother–child dyads withdrew or were excluded (reported previously, [20]). As mentioned earlier, in the current study, mother–child dyads were included in the analysis if they had used the accelerometer as instructed during the baseline week and the final intervention week and if the mother answered the enjoyment questions at baseline as well as the end. Acceptable accelerometer data were available for 121 mother–child dyads and 140 mothers answered the enjoyment questions. Looking at the criteria together, data were found altogether for 53% of 203 mother–child dyads originally included in the Moving Sound RCT analysis.

Compared to the dyads that were excluded (*n* = 95), children among the dyads who were included (*n* = 108) were more likely to be in daycare or preschool for at least 3 days per week (94% vs. 75%, *p* < 0.001). There were no statistical differences between these two groups in belonging to the intervention or control group, mothers’ working status, gender of the participating child, age or the BMI of mothers or children, number of children in the family, mothers’ perceived health, or levels of SB or PA during the baseline week.

## Discussion

The present study aimed to assess the relationship between a movement-to-music video exercise program in the home environment and mothers’ enjoyment of exercise with their child for 8 weeks of intervention. The study also reported the relationship between mothers’ enjoyment and device-based measurements of children’s and mothers’ SB and PA, as well as adherence to the training program.

### Mothers’ enjoyment of exercise with their child

The present analyses demonstrated that most mothers showed a stable enjoyment score during the eight-week intervention. Mothers in the intervention group received the movement-to-music video for exercising with their child at the beginning of the second week. Overall, the baseline of the mothers’ enjoyment scores was high in both groups. The result indicated that a roof-effect restricted an increase of enjoyment scores in both the intervention and control group. However, on average, mothers in the intervention group increased their enjoyment scores over the study period. We assume that exercising together with the child strengthens the relationship between the mother and child through positive experiences, and could thereby increase the mother’s enjoyment of exercise with the child. This is partly in line with Hallam [19], who observed that shared musical activities may benefit mother–child interactions and communications. These intrinsically rewarded experiences may increase both mothers’ and children’s enjoyment.

An understanding of the influence of enjoyment is important for the design and implementation of new studies. Precise goal setting might be the additional impetus for busy parents to prioritize their children’s PA above other competing demands [28]. Thus, using the behavioral change models, it would be possible to take into account, for example, capability, opportunity, and motivation, which are all determinants of health behavior [29].

### Relationships between exercises with the video program and mothers’ enjoyment

Within the intervention group, the level of exercise adherence was low during the final intervention week (results reported earlier) [20, 21], being around 20% in this sample. As mothers presented a roof-effect in enjoyment scores at the baseline, the issue was particularly visible between those participants who used the video during the last week compared to those who did not. Even if mothers’ enjoyment was on average higher at the end in both the adherent and non-adherent groups, we believe that this test (meaning EIS questionnaire) might not be sensitive enough to detect changes in mothers’ enjoyment in the short term.

Information on the number of exercises performed with a movement-to-music video was collected via exercise diaries. This leads to the question of whether the mother–child dyads with mothers’ increased enjoyment have found some other ways to play or exercise together. Mothers were asked to record their own and their child’s daily exercise sessions in the diaries, but we do not know how much mothers and children exercised together. Thus, it is reasonable to ask whether the participants performed the exercises only for study purposes, not for playing or having fun together.

Scanlan et al. [11] defined enjoyment as a positive affective response to the sport experience. Based on previously published comments by the the mothers and children [21], it seemed that most mothers and children got tired of the sameness in the exercise program, so the exercise adherence was low. The importance of changes within exercise performance, the possibility to do more challenging movements and have an additional load as the intervention progresses should be considered in future studies to maintain enjoyment. The mothers’ role as a facilitator is also important: in the current study, most of the exercises were meant to be done together. Some of the mother–child dyads who used the video during the final week reported that the whole family performed the exercises together, along with the mother and child.

### Relationship between mothers’ baseline enjoyment and PA and SB

Mothers’ enjoyment at baseline was not related to their own and their children’s PA, and SB did not reach the level of statistical significance between the groups of higher or lower enjoyment. Our findings contrast with Solomon-Moore et al. [3], who found that parents’ intrinsic motivation, including enjoyment, and intention to engage in regular family-based PA was positively associated with parents’ MVPA. Regarding children, their finding that parents’ intrinsic motivation was positively associated with children’s MVPA was not supported by our results. Children’s own intrinsic motivation has also been found to be a significant predictor for both PA enjoyment and MVPA in children, especially among 8- to 14-years-olds [30]. However, it was not studied in this research.

A direct association has been reported between maternal role modeling and MVPA among boys and maternal co-participation and MVPA among girls [31]. One of the best predictors of children’s higher MVPA on weekdays has been greater time spent by mothers in organized PA with children, while during weekends, the father’s role was more important than the mother’s [2]. To our knowledge, parents’ role modeling and co-participation are partly influenced by motivation and enjoyment. In this study, we studied the mothers’ enjoyment of exercise with their child instead of co-exercising itself. Besides, we did not separate weekdays and weekends, and the focus of the study was on mother–child dyads instead of the family. In this study, children’s LPA increased if their mothers had higher enjoyment of exercising with them, and children’s SB increased if their mothers had lower enjoyment. However, as shown in Fig. 2, an increase in SB seemed to take the place mostly of standing and an increase in LPA from MVPA, which are both undesirable changes. These results are partly in line with Remmers et al. [12], who found that enjoyment of PA was related to active behavior. Cantell et al. (2012) also concluded that parental involvement in PA with their children appeared to promote higher levels of MVPA in children [2]. For future studies, it would be important to characterize not only the effect of enjoyment but also the capability and opportunities for explaining the reasons behind the behavior changes [29].

### Relationships between changes in mothers’ enjoyment and PA and SB

Although there was no difference between the intervention and control groups, mothers in the intervention group increased their enjoyment during the intervention. It is notable that if mothers’ enjoyment decreased, their own LPA and their children’s SB increased over time. These changes may be related to the high baseline level of mothers’ enjoyment, as well as lower levels of LPA and SB at baseline compared to other enjoyment groups. We also found that if mothers’ enjoyment remained stable, the children increased their LPA and Total PA. If mothers increased their enjoyment, changes in their children’s PA or SB were not found. This result suggests that an increase in mothers’ enjoyment of exercise with the child does not automatically change mothers or their children’s PA or SB.

Parents’ external control for children may be associated negatively with the child’s PA [3], which might be a case in these kinds of studies, specifically if a parent does not exercise together with a child. Though speculative, and thus requiring additional research to confirm, we assume that mothers’ (free) play with their children is likely to improve the relationship between mothers and children and thereby increase the mothers’ enjoyment of moving and exercising with their children.

Small PA equipment, such as a pedometer or other wearable devices, might make parents and children more aware of their PA levels [32]. In this study, mother–child dyads received their accelerometer results after the intervention period, but it is possible that knowing they were being measured might have influenced the participants’ behavior by increasing PA at the beginning of the study.

### Strengths and limitations

The major strength of the study is the RCT design and the use of the feasible [25–27] tri-axial accelerometer for measurements. The enjoyment questionnaire has been developed to measure enjoyment in sport [11, 23], and the modified version to measure enjoyment of exercising with the child [22] was piloted.

Because of the multi-dimensional and complex nature of behavior change [4, 29], there are several limitations in the current study. The number of the mother–child dyads with acceptable accelerometer measurements and who answered the questions about mothers’ enjoyment of exercise was lower than expected: 228 mother–child dyads were recruited for the RCT, and 108 of them met both of the prerequisites set for this study. The sample size regarding different groups was therefore smaller than it should have been according to power calculations. For this reason, part of the results might show no differences between groups due to a lack of statistical power, and the results can be considered as indicative only.

Furthermore, the current study was a short-term intervention, and maintenance of behavior change over the longer term was not assessed. With regard to children’s and parent’s SB and PA, more high-quality long-term RCTs are needed to examine the effect of enjoyment.

O’Connor et al. [33] concluded that educational or training program interventions, which include family visits or telephone communication with parents, are promising for the promotion of PA. Since we know how important a role motivation plays in decreasing SB and increasing PA, the movement-to-music video program used in the present study may have been too repetitive. We also did not have any visits or communication with families during the intervention. This may have influenced the exercise enjoyment and adherence of the mother–child dyads.

We also studied the relationship between mothers’ enjoyment and mothers’ and children’s SB and PA. It is known that support from parents and their activity as role models is related to children’s PA [34]. To our knowledge, there is growing interest in exercise interventions focused on the SB and PA of parents and 5- to 7-year old children. However, when enjoyment has been included, the main focus has been how to make exercises enjoyable for children.

In family studies, it would be essential to study the effect of both parents on children. However, we did not study the relationship between fathers and children. Cantell et al. (2012) found that mothers’ role as an enabler is more influential during weekdays and fathers’ during weekends. In the current study, we did not separate weekdays or weekends, or the time mother–child dyads spent together from daycare or preschool and working time. We analyzed the proportions of SB and PA during mothers’ and children’s waking time. The result was that the percentage using video programs in the home environment was smaller than if analyzed as a part of mother–child dyads’ leisure time. This choice may have influenced the results.

PA and SB, as measured by the accelerometer, described overall movement and activity during waking hours, but it did not indicate where and with whom that movement was done. Thus, the total PA and SB might describe something different from exercise according to the video.

Several sets of tests were run for both mothers and children. This can cause false positives in results since corrections related to the number of tests and significance were not done.

Despite these limitations, the current study provides valuable information about mothers’ enjoyment of exercise with their children and the effects of performed exercises on children’s SB and PA.

## Conclusions

This study provided information on the relationship between mothers’ enjoyment of a movement-to-music exercise intervention and PA and SB within mother–child dyads. Mothers’ enjoyment of exercise with their children was overall high. Mothers in the intervention group scored higher on the enjoyment scale at the end of the study than they did at the baseline. However, the difference between the intervention and the control group was not statistically significant. Children with mothers who scored higher in enjoyment at the baseline increased their LPA during the study. Children with mothers who scored lower in enjoyment increased their SB. Further, children’s SB increased if their mothers’ enjoyment decreased, and children’s LPA and Total PA increased if their mothers’ enjoyment remained stable. A relationship between the use of the video program and the changes in mothers’ enjoyment differed between mothers and children. These results highlight the complicated nature of enjoyment in PA behavior. For future studies, it would be important to focus on the whole family and take into account children’s own enjoyment, motivation, and factors behind the behavior change.

## Supplementary Information

**Additional file 1.**

**Additional file 2.**

## Data Availability

Study participants did not consent to have their data publicly available. Music and video content are protected by copyright laws. Due to ethical restrictions of the local Ethics Committee, data are available from the UKK Institute of Health Promotion Research, Tampere, Finland, for researchers who meet the criteria for access to confidential data. For data requests and permissions, contact tiedotus@ukkinstituutti.fi.

## Abbreviations

EIS: Enjoyment in sport
LPA: Light physical activity
MVPA: Moderate-to-vigorous physical activity
PA: Physical activity
RCT: Randomized controlled trial
SB: Sedentary behavior, including lying, reclining, and sitting
SD: Standard deviation
SS: standing
Total PA: Total physical activity, including light, moderate, and vigorous physical activity
