# Supplementary material for: Relationship between mothers’ enjoyment and sedentary behavior and physical activity of mother–child dyads using a movement-to-music video program: a secondary analysis of a randomized controlled trial
Source: BMC Public Health. 2020 Nov 4;20:1659. doi: 10.1186/s12889-020-09773-4 (PMC7640412; doi:10.1186/s12889-020-09773-4)
Supplement: Supplementary file 1 — Additional file 1. [file 12889_2020_9773_MOESM1_ESM.docx]

Supplementary Table 1. Differences at the baseline and change within and between the enjoyment groups of mothers in sedentary behavior and physical activity over time as a proportion of measurement time (estimates, 95% confidence intervals (CI), and *p* value from the non-adjusted linear mixed-effects model).

| Mothers (n = 108) | | Estimate (95% CI) | *p*-value |
| --- | --- | --- | --- |
| Sedentary behavior | |  |  |
|  | Enjoyment stayed stable (ref = decreased) | -1.10 (-6.17 to 3.97) | 0.67 |
|  | Enjoyment increased (ref = decreased) | 1.43 (-3.88 to 6.73) | 0.60 |
|  | Change in time, decreased | -0.20 (-0.64 to 0.25) | 0.39 |
|  | Change in time, stayed stable | 0.01 (-0.26 to 0.29) | 0.92 |
|  | Change in time, increased | -0.07 (-0.39 to 0.24) | 0.64 |
|  | Intervention effect, stayed stable (ref = decreased) | 0.21 (-0.31 to 0.73) | 0.43 |
|  | Intervention effect, increased (ref = decreased) | 0.12 (-0.42 to 0.67) | 0.66 |
| Standing | |  |  |
|  | Enjoyment stayed stable (ref = decreased) | -0.57 (-3.39 to 2.25) | 0.69 |
|  | Enjoyment increased (ref = decreased) | -1.55 (-4.50 to 1.40) | 0.30 |
|  | Change in time, decreased | -0.08 (-0.37 to 0.21) | 0.58 |
|  | Change in time, stayed stable | -0.03 (-0.21 to 0.15) | 0.76 |
|  | Change in time, increased | -0.01 (-0.22 to 0.20) | 0.93 |
|  | Intervention effect, stayed stable (ref = decreased) | 0.05 (-0.29 to 0.40) | 0.76 |
|  | Intervention effect, increased (ref = decreased) | 0.07 (-0.28 to 0.43) | 0.69 |
| Light physical activity | |  |  |
|  | Enjoyment stayed stable (ref = decreased) | 1.33 (-0.99 to 3.66) | 0.26 |
|  | Enjoyment increased (ref = decreased) | -0.01 (-2.44 to 2.43) | 1.00 |
|  | Change in time, decreased | 0.19 (0.001 to 0.39) | **0.049** |
|  | Change in time, stayed stable | 0.01 (-0.10 to 0.13) | 0.86 |
|  | Change in time, increased | 0.06 (-0.07 to 0.19) | 0.39 |
|  | Intervention effect, stayed stable (ref = decreased) | -0.18 (-0.40 to 0.04) | 0.11 |
|  | Intervention effect, increased (ref = decreased) | -0.13 (-0.36 to 0.10) | 0.68 |
| Moderate-to-vigorous physical activity | |  |  |
|  | Enjoyment stayed stable (ref = decreased) | 0.34 (-1.32 to 2.00) | 0.69 |
|  | Enjoyment increased (ref = decreased) | 0.13 (-1.60 to 1.87) | 0.88 |
|  | Change in time, decreased | 0.09 (-0.06 to 0.24) | 0.25 |
|  | Change in time, stayed stable | 0.00 (-0.09 to 0.10) | 0.94 |
|  | Change in time, increased | 0.02 (-0.08 to 0.13) | 0.65 |
|  | Intervention effect, stayed stable (ref = decreased) | -0.09 (-0.26 to 0.09) | 0.35 |
|  | Intervention effect, increased (ref = decreased) | -0.06 (-0.25 to 0.12) | 0.50 |
| Total physical activity | |  |  |
|  | Enjoyment stayed stable (ref = decreased) | 1.67 (-1.83 to 5.17) | 0.35 |
|  | Enjoyment increased (ref = decreased) | 0.13 (-3.54 to 3.79) | 0.95 |
|  | Change in time, decreased | 0.28 (-0.01 to 0.56) | 0.055 |
|  | Change in time, stayed stable | 0.01 (-0.16 to 0.19) | 0.88 |
|  | Change in time, increased | 0.08 (-0.12 to 0.28) | 0.42 |
|  | Intervention effect, stayed stable (ref = decreased) | -0.26 (-0.60 to 0.07) | 0.12 |
|  | Intervention effect, increased (ref = decreased) | -0.19 (-0.54 to 0.15) | 0.27 |
