# Supplementary material for: Relationship between mothers’ enjoyment and sedentary behavior and physical activity of mother–child dyads using a movement-to-music video program: a secondary analysis of a randomized controlled trial
Source: BMC Public Health. 2020 Nov 4;20:1659. doi: 10.1186/s12889-020-09773-4 (PMC7640412; doi:10.1186/s12889-020-09773-4)
Supplement: Supplementary file 2 — Additional file 2. [file 12889_2020_9773_MOESM2_ESM.docx]

Supplementary Table 2. Differences at the baseline and change within and between the groups of children in sedentary behavior and physical activity over time as a proportion of measurement time (estimates, 95% confidence intervals (CI), and *p* value from the non-adjusted linear mixed-effects model). Groups divided by changes in mothers' enjoyment.

| Children (n = 108) | | Estimate (95% CI) | *p*-value |
| --- | --- | --- | --- |
| Sedentary behavior | |  |  |
|  | Enjoyment stayed stable (ref = decreased) | 0.90 (-2.61 to 4.41) | 0.62 |
|  | Enjoyment increased (ref = decreased) | 3.46 (-0.21 to 7.13) | 0.065 |
|  | Change in time, decreased | 0.52 (0.11 to 0.93) | **0.013** |
|  | Change in time, stayed stable | 0.10 (-0.15 to 0.35) | 0.44 |
|  | Change in time, increased | 0.11 (-0.18 to 0.40) | 0.45 |
|  | Intervention effect, stayed stable (ref = decreased) | -0.42 (-0.90 to 0.06) | 0.085 |
|  | Intervention effect, increased (ref = decreased) | -0.41 (-0.91 to 0.09) | 0.11 |
| Standing | |  |  |
|  | Enjoyment stayed stable (ref = decreased) | 0.33 (-1.05 to 1.71) | 0.64 |
|  | Enjoyment increased (ref = decreased) | -0.32 (-1.76 to 1.12) | 0.66 |
|  | Change in time, decreased | -0.06 (-0.22 to 0.10) | 0.44 |
|  | Change in time, stayed stable | 0.00 (-0.09 to 0.10) | 0.92 |
|  | Change in time, increased | -0.02 (-0.13 to 0.09) | 0.72 |
|  | Intervention effect, stayed stable (ref = decreased) | 0.07 (-0.12 to 0.25) | 0.48 |
|  | Intervention effect, increased (ref = decreased) | 0.04 (-0.15 to 0.24) | 0.67 |
| Light physical activity | |  |  |
|  | Enjoyment stayed stable (ref = decreased) | -0.16 (-1.97 to 1.64) | 0.86 |
|  | Enjoyment increased (ref = decreased) | -0.12 (-2.00 to 1.77) | 0.90 |
|  | Change in time, decreased | 0.08 (-0.14 to 0.32) | 0.47 |
|  | Change in time, stayed stable | 0.22 (0.08 to 0.36) | **0.002** |
|  | Change in time, increased | 0.15 (-0.01 to 0.32) | 0.064 |
|  | Intervention effect, stayed stable (ref = decreased) | 0.14 (-0.13 to 0.41) | 0.31 |
|  | Intervention effect, increased (ref = decreased) | 0.07 (-0.21 to 0.35) | 0.63 |
| Moderate-to-vigorous physical activity | |  |  |
|  | Enjoyment stayed stable (ref = decreased) | -1.16 (-3.37 to 1.04) | 0.30 |
|  | Enjoyment increased (ref = decreased) | -2.45 (-4.76 to -0.14) | **0.037** |
|  | Change in time, decreased | -0.21 (-0.45 to 0.03) | 0.091 |
|  | Change in time, stayed stable | 0.02 (-0.13 to 0.17) | 0.78 |
|  | Change in time, increased | 0.01 (-0.16 to 0.18) | 0.93 |
|  | Intervention effect, stayed stable (ref = decreased) | 0.23 (-0.05 to 0.51) | 0.11 |
|  | Intervention effect, increased (ref = decreased) | 0.22 (-0.08 to 0.51) | 0.15 |
| Total physical activity | |  |  |
|  | Enjoyment stayed stable (ref = decreased) | -1.33 (-4.48 to 1.83) | 0.41 |
|  | Enjoyment increased (ref = decreased) | -2.57 (-5.87 to 0.73) | 0.13 |
|  | Change in time, decreased | -0.12 (-0.50 to 0.25) | 0.52 |
|  | Change in time, stayed stable | 0.25 (0.02 to 0.47) | **0.034** |
|  | Change in time, increased | 0.16 (-0.10 to 0.42) | 0.23 |
|  | Intervention effect, stayed stable (ref = decreased) | 0.37 (-0.07 to 0.80) | 0.097 |
|  | Intervention effect, increased (ref = decreased) | 0.28 (-0.17 to 0.74) | 0.22 |
